# Acute Pancreatitis Caused by Complications of Influenza A in the Setting of Chronic Lymphocytic Leukemia

**DOI:** 10.7759/cureus.7067

**Published:** 2020-02-21

**Authors:** Cesar Avalos, Elias Estifan, Shelbi Swyden, Ruhin Yuridullah

**Affiliations:** 1 Internal Medicine, St. Joseph's University Medical Center, Paterson, USA; 2 Internal Medicine, St. George's University, True Blue, GRD; 3 Internal Medicine, St. Joseph's Univeristy Medical Center, Paterson, USA

**Keywords:** acute pancreatitis, influenza a, chronic lymphocytic leukemia

## Abstract

Although most cases of acute pancreatitis are attributed to alcohol and gallstones, acute pancreatitis can be a presenting feature or complication of a viral etiology (influenza). We report a rare case of acute pancreatitis secondary to H1N1 influenza A virus in the setting of chronic lymphocytic leukemia. The typical flu-like respiratory illness usually observed with influenza was absent preceding the episode of pancreatitis owing to the different antigenic properties of influenza A (compared to influenza B) and an underlying immunocompromised state.

## Introduction

Acute pancreatitis (AP) is caused by an activation of pancreatic enzymes, which causes inflammation and interstitial edema of the pancreas. The common causes include biliary outflow obstruction secondary to gallstones, alcohol consumption, and medications. Other etiologies include hypertriglyceridemia, trauma, iatrogenic, hypercalcemia, and viral (HIV, hepatitis B, mumps, cytomegalovirus, herpes simplex) induced [1]. Viral illness rarely causes pancreatitis, and the H1N1 influenza virus has only been documented in a handful of occasions as the etiology for AP [2]. Recent studies have shown that the influenza A virus is able to bind to human pancreatic cells and replicate, inducing apoptosis and triggering the release of proinflammatory cytokines and chemokines [3]. The tropism for human pancreatic cell lines establishes a likely etiology for influenza A to induce AP [3]. 

## Case presentation

An 86-year-old male with a past medical history of untreated chronic lymphocytic leukemia (CLL), Alzheimer’s disease, coronary artery disease, hypothyroidism, atrial fibrillation, and depression presented to the emergency department (ED) from a long-term nursing facility after sustaining a fall to the ground after attempting to stand from a seating position. An hour prior to the fall, he had experienced sudden onset of stabbing epigastric abdominal pain, nonradiating, 7/10 in severity with associated nausea without any other prodromal symptoms. Upon arrival at the ED, he began to vomit large quantities of partially digested food products without any blood or bile. Past surgical history consisted of prior cholecystectomy. There was no history of previous similar complaints, recent travel, alcohol consumption, tobacco, or drug use, and he was not on any treatment for CLL. He endorsed being in close contact with other residents at the nursing home suffering from an upper respiratory infection.

The initial vital signs in ED were as follows: blood pressure (BP) 147/92 mmHg, heart rate 79 beats per minute with a normal rhythm, respiratory rate of 16 breaths per minute, oxygen saturation 99% on room air, and temporal artery temperature of 36.3 degrees Celsius. On physical examination, a frail gentleman with a 2 cm linear laceration overlying the lateral aspect of the right eyelid and small amount of bleeding was sitting upright, awake, alert, and in no acute distress. The abdominal exam revealed mild epigastric tenderness without distension, guarding, rebound, and negative Murphy’s sign. Cardiovascular and pulmonary examinations were unremarkable. No additional traumatic lesions were noted on examination; all other systems were grossly normal. Laboratory studies revealed elevated random blood glucose of 133 mg/dL, and a complete blood count showed results consistent with known CLL with a total leukocyte count (lymphocyte-predominant) of 50x10^3^/mm^3^ (reference range: 4.5-11.0^3^/mm^3^) and elevated levels of atypical lymphocytes. Platelet was 227x10^9^/L, hemoglobin 12.1, and packed cell volume 36.5%. Additional laboratory studies, including liver function, cholesterol panel, and serum chemistry (except creatinine level of 1.15 mg/dL), were within the normal reference range. Serum lipase was 1,406 U/L (reference range: 0-100 U/L), and a throat swab for reverse transcriptase PCR was positive for the H1N1 influenza virus. HIV was nonreactive.

**Figure 1:**
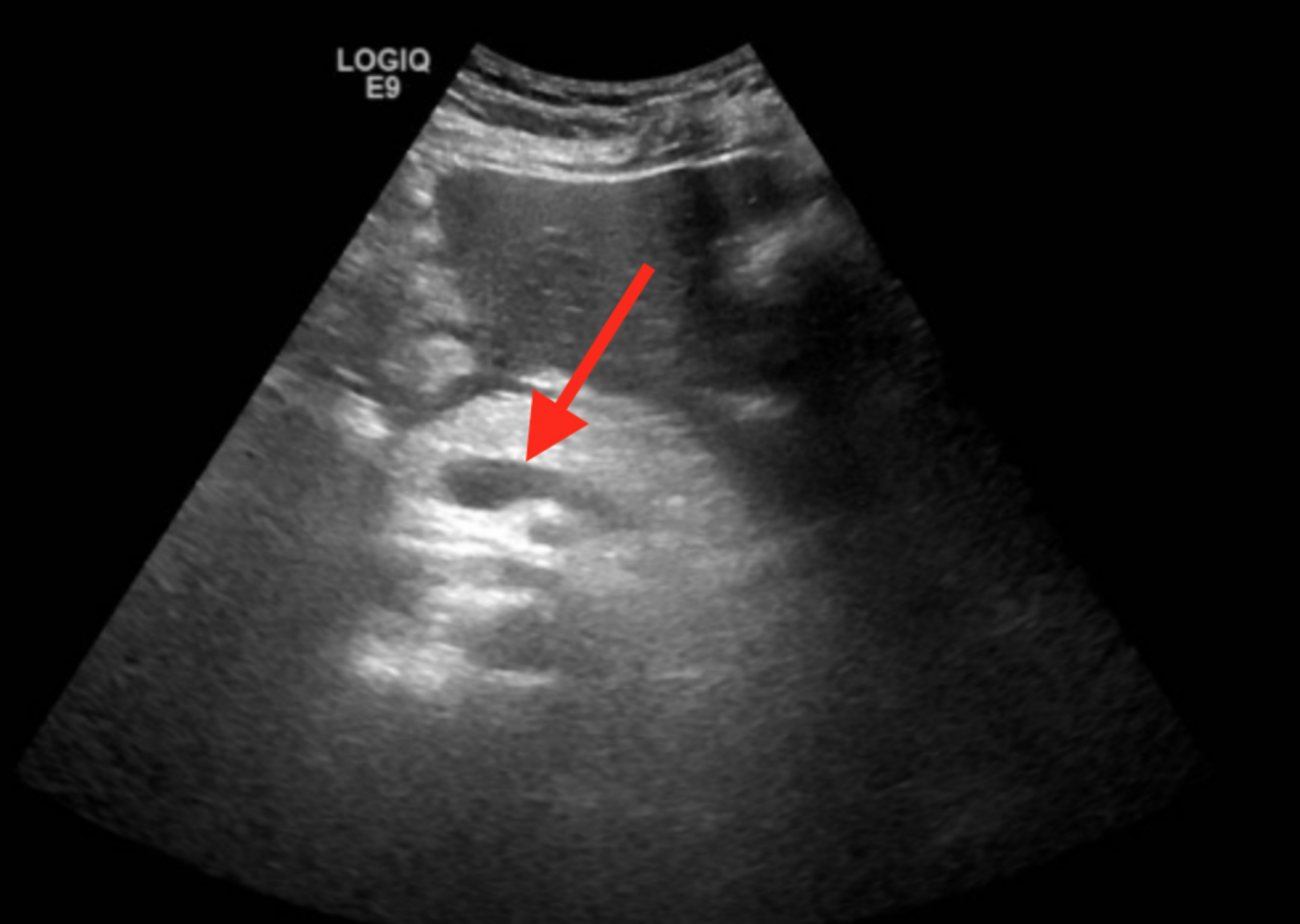
Ultrasound of the abdomen Cystic structure in the head of the pancreas measuring 1.7 x 1.4 x 1.4 cm (arrow), pancreas prominent with inhomogeneous ultrasonic morphology consistent with pancreatitis

Chest radiograph revealed no infiltrates, effusion, or pneumothorax except for an elevated left hemidiaphragm and atelectasis at the left base, unchanged from prior studies. Noncontrast enhanced CT scan of the head was performed due to the fall and facial laceration, which revealed no acute intracranial hemorrhage, midline shift, or mass effect. Ultrasound of the abdomen demonstrated a cystic structure in the head of the pancreas measuring 1.7 x 1.4 x 1.4 cm; the pancreas was prominent with inhomogeneous ultrasonic morphology consistent with pancreatitis (Figure 1). No peripancreatic fluid was seen on ultrasound, and the common bile duct measured 2.7 mm with a surgically absent gallbladder. A contrast-enhanced CT scan of the abdomen was deferred, given the acute renal insufficiency on presentation.

During the initial hospital course, he was febrile (Tmax 38.5 degrees Celsius), remained normotensive (BP 120/60 mmHg), but tachycardic (110 beats per minute). He was started on intravenous fluid therapy with normal saline, acetaminophen for pain control, and oseltamivir orally. Apart from the first day, he remained afebrile, responded well to the initial conservative therapy, and blood and urine cultures remained negative. Diet was advanced to solid foods on day 2, which he tolerated, and he was discharged home in stable condition on a five-day course of oseltamivir. He had remained symptom-free at the four-week follow-up appointment.

## Discussion

AP results from the inflammation of the pancreas most commonly secondary to gallstones and alcohol abuse, but also due to a variety of other factors such as elevated triglycerides and calcium levels, drugs, trauma, and post-endoscopic retrograde cholangiopancreatography [1,4]. Less commonly, viral illnesses such as mumps, HIV, coxsackie, hepatitis B, cytomegalovirus, and herpes simplex have been noted to cause pancreatitis as the risk increase in immunocompromised patients [1,2,4,5]. However, viral influenza (H1N1) has rarely been reported to cause AP. There are only five to six cases of H1N1 influenza, causing AP to have been reported in the literature [4,5-7].

Evidence for influenza causing AP and diabetes comes from in vitro and in vivo studies [3,8]. Influenza viruses replicate only in the presence of trypsin or trypsin-like enzymes, and thus their replication is believed to be restricted to the respiratory and enteric tracts [6]. Postmortem pancreatic lesions from birds, cats, and dogs after influenza infection have shown findings ranging from inflammation to pancreatic necrosis [8]. In vitro and in vivo mouse models cytokine expression profile of the H1N1 viruses revealed that they could cause proinflammatory response in pancreatic islets given the fact that IFN-gamma-inducible chemokines IP-10/CXCL10 and MIG/CXCL9 were at the highest level after infection [3,8].

In the case of our patient, influenza A infection likely lead to the onset of AP. Typical flu-like upper respiratory symptoms consistent with influenza were absent preceding the episode of pancreatitis owing likely to the different antigenic properties of influenza A (compared to influenza B) and an underlying immunocompromised state. He was not on any chemotherapeutic agents, which could have confounded the findings of pancreatitis, and he denied any alcohol consumption or any history of gallstones. Furthermore, some in vitro, in vivo, and case reports have correlated H1N1 influenza infection with an onset of diabetes post-infection due to the ability of the virus to replicated in pancreatic beta-cells [9-12].

## Conclusions

We present a rare case of influenza A causing AP in an adult suffering from CLL. Although rare, adding influenza as yet another cause of AP and possibly playing a role in the etiopathogenesis of diabetes in humans would further add to the importance of seasonal vaccination against influenza.
